# Retinal primary cilia and their dysfunction in retinal neurodegenerative diseases: beyond ciliopathies

**DOI:** 10.1186/s10020-024-00875-y

**Published:** 2024-07-26

**Authors:** Xiaonan Liu, Anna Pacwa, Giorgia Bresciani, Marta Swierczynska, Mariola Dorecka, Adrian Smedowski

**Affiliations:** 1 Department of Ophthalmology, Faculty of Medical Sciences in Katowice, Medical University of Silesia, Ceglana 35, 40-514, Katowice, Poland; 2 Institute of Biotechnology, HiLIFE, University of Helsinki, Helsinki, Finland; 3 GlaucoTech Co, Katowice, Poland; 4 Department of Drug Sciences, University of Pavia, Pavia, Italy; 5 Department of Ophthalmology, Professor K. Gibinski University Clinical Center, Medical University of Silesia, Katowice, Poland; 6 Department of Ophthalmology, Faculty of Medical Sciences in Katowice, Medical University of Silesia, Ceglana 35, 40-514, Katowice, Poland; 7 Department of Physiology, Faculty of Medical Sciences in Katowice, Medical University of Silesia, Medykow 18, 40-752 Katowice, Poland

**Keywords:** Retinal ciliopathy, Retinitis pigmentosa, Retinal dystrophy, Photoreceptors, RGC cells, Inherited blindness

## Abstract

Primary cilia are sensory organelles that extend from the cellular membrane and are found in a wide range of cell types. Cilia possess a plethora of vital components that enable the detection and transmission of several signaling pathways, including Wnt and Shh. In turn, the regulation of ciliogenesis and cilium length is influenced by various factors, including autophagy, organization of the actin cytoskeleton, and signaling inside the cilium. Irregularities in the development, maintenance, and function of this cellular component lead to a range of clinical manifestations known as ciliopathies. The majority of people with ciliopathies have a high prevalence of retinal degeneration. The most common theory is that retinal degeneration is primarily caused by functional and developmental problems within retinal photoreceptors. The contribution of other ciliated retinal cell types to retinal degeneration has not been explored to date. In this review, we examine the occurrence of primary cilia in various retinal cell types and their significance in pathology. Additionally, we explore potential therapeutic approaches targeting ciliopathies. By engaging in this endeavor, we present new ideas that elucidate innovative concepts for the future investigation and treatment of retinal ciliopathies.

**Supplementary Information:**

The online version contains supplementary material available at 10.1186/s10020-024-00875-y.

## Introduction

**Fig. 1:**
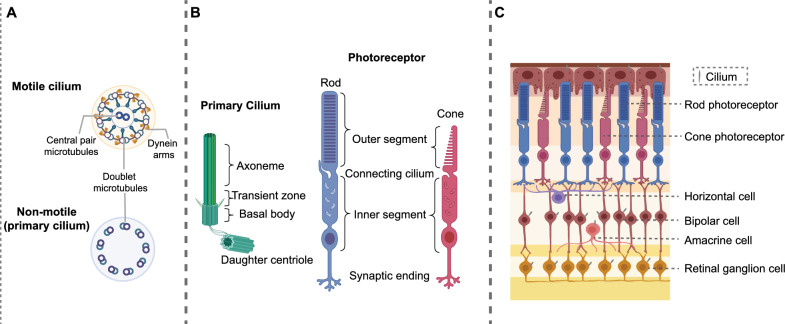
Primary cilia in the neurosensory retina layers. **A** Diagrams show views of motile (9 + 2) and nonmotile (9 + 0) cilia in cross section. **B** The architecture of the general primary cilium (left) and the photoreceptor cilium (right). **C** The multilayered structure of the retina with primary cilia

Cilia are membrane-bound, antenna-like organelles that protrude from the cell surface, with widespread occurrence across several cell types and crucial involvement in both physiological development and genetic disorders (Pala et al. 2017). Cilia can be classified as motile or nonmotile (Fig. 1A); both types share a basic ciliary structure, consisting of an axoneme made of nine microtubule doublets extending from the basal body (BB), rooted in a “microtubular organizing center” (MTOC) (Sanchez and Feldman 2017) and attached to the cell membrane by transition fibers (Horani and Ferkol 2021). The ciliary membrane is continuous with the plasma membrane but contains a unique protein composition of channels and receptors (Pala et al. 2017). Outside of this common basic structure, differences are also present between the two types of cilia in both structure and function; compared to motile cilia, immotile primary cilia lack the two central microtubules and only have nine microtubule doublets at the edge of the axonemes (Fig. 1A).

Primary cilia have a sensory role (mechanosensors, chemosensors or neurosensors) and are rich in moieties essential for transducing and regulating a variety of signaling cascades, including the Wnt, Sonic Hedgehog (SHH), Notch, platelet-derived growth factor (PDGF), and mammalian target of rapamycin (mTOR) pathways, in most cells (Alhashem et al. 2022; Mill et al. 2023). This highly organized structure has also been co-opted by major sensory neurons, such as olfactory cells and retinal photoreceptors. Photoreceptor cells in the retina have a specialized structure called the outer segment, commonly referred to as a modified cilium (Fig. 1B). Unlike the primary cilia found in most cells, which can appear and disappear at specific stages of the cell cycle, the specialized cilia of photoreceptor cells are unique in that they are continuously present and undergo constant renewal to meet the ongoing demands of visual perception.

The spectrum of clinical manifestations caused by abnormalities in the formation, upkeep, and function of these organelles are collectively referred to as ciliopathies (Anvarian et al. 2019). The near ubiquity of these organelles across cell types and their fundamental role in development are responsible for the prominent phenotypes associated with the central nervous system displayed in ciliopathies; moreover, the importance of primary cilia function within the eye is particularly evident from the variety of retinal symptoms, e.g., retinal degeneration, retinitis pigmentosa, rod-cone dystrophies, and optic neuropathies (Chen et al. 2019; Ning et al. 2023). Currently, the majority of research on the involvement of cilia in these symptoms focuses on photoreceptor cells, but primary cilia have also been found in other types of neurons in the retina (Ning et al. 2021; Sun et al. 2021; Lepanto et al. 2016a, b). Recent studies have expanded to elucidate the role of primary cilia in other parts of the eye, but their functions and the stages of development during which these organelles are exhibited remain unclear. An additional challenge is presented by the visualization of primary cilia in retinas, due to their dimensions and intricate composition and the constraints imposed by existing imaging methodologies (Yang et al. 2016; Filipova et al. 2020; Li et al. 2022).

Recent proteomic investigations have significantly enhanced our understanding of the proteins involved in the retina, particularly those associated with primary cilia. Studies focusing on inherited retinal dystrophies and other ciliopathies have identified hundreds of unique proteins that form integral components of primary sensory cilia. A thorough proteomic investigation of mouse photoreceptor cilia discovered 2000 proteins overall, hundreds of which are also found in other types of cilia (Liu et al. 2007). This discovery not only underscores the complexity and diversity of the protein composition in retinal cilia but also highlights their fundamental similarities to cilia in other cellular contexts.

In this review, we discuss recent evidence on the role of cilia in different retinal neuronal cells and then focus on our present comprehension of retinal neurodegenerative diseases associated with ciliary abnormalities. We seek to elucidate the regulatory mechanisms of cilia formation, as well as the specific roles that cilia play in retinal neurodegenerative diseases. In addition to sharing current information, we attempt to identify gaps in our understanding of cilia biology and propose techniques for future research.

## Primary cilia in neurosensory retina layers

The retina is highly complex syncytial structures in vertebrate, and is responsible for processing the visual impulses that generate in photoreceptor cells through phototransduction. The visual information is then processed by bipolar and ganglion cells, and modulated by interneurons such as amacrine and horizontal cells. It is then transmitted through the optic nerve to the primary and secondary visual centers in the brain, located in the lateral geniculate nucleus and the occipital cortex, respectively (Fig. 1C). Dysfunctions at any level can lead to a variety of pathologies, including ciliopathies, which are among the major causes of irreversible blindness.

### Retinal ganglion cells (RGCs)

Retinal ganglion cells (RGCs) are located in the innermost layer of the retina (Fig. 1C), specifically referred to as the ganglion cell layer. RGCs are the first retinal neurons to be formed during development in all vertebrates, after which the remaining retinal cell types are generated (Bassett and Wallace 2012; Centanin and Wittbrodt 2014; Hu and Easter 1999; Stenkamp 2015). More specifically, the central projections of the vertebrate retina originate from RGCs that express the basic helix-loop-helix (bHLH) gene Atoh7 (Fritzsch and Martin 2022); in fact, bHLH genes and their orthologs [e.g., achaete-scute family bHLH transcription factor 1 (ASCL1), atonal bHLH transcription factor 1 (ATOH1/7), neurogenin 1/2 (Neurog1/2), and neuronal differentiation 1 (Neurod1)] are essential for the development of approximately 90% of neurons and sensory cells in the vertebrate brain, eye and ear (Fritzsch 2021; Fritzsch and Martin 2022; Fritzsch et al. 2014).

Research from the early 1980s demonstrated that a majority of RGCs possess primary cilia (Boycott and Hopkins 1984). It has been documented that primary cilia in RGCs are crucial for various aspects of RGC function, including differentiation, developmental processes, and the formation of central projections. Notably, they might also play a key role in promoting axon regeneration in adult RGCs.

In vivo, the differentiation of RGCs is meticulously orchestrated by the interplay of cell-specific transcription factors and signaling factors derived from the surrounding tissue. The primary cilium, acting as a central signaling hub within the cell, is crucial during the generation and differentiation of RGCs, though its specific roles and dynamics have yet to be comprehensively evaluated in vivo. A study focusing on retinal neuroepithelial cells discovered that these cells typically feature a primary cilium located at the apical membrane and extending outward, and additionally, a small subset of cilia is found beneath the apical region both prior to and during the neurogenic phase (Lepanto et al. 2016a, b). During apical process retraction and dendritogenesis, this organelle remains positioned apically in neuroblasts. However, between these stages, cilia exhibit considerable dynamism in terms of their presence and position. Disrupting cilia function results in a reduction in the proliferation of retinal progenitor cells and a decrease in neural retina volume. Furthermore, retinal histogenesis is generally delayed, with a particular impact on the formation of the RGC layer compared to the amacrine and photoreceptor cell layers. The abovementioned polarized primary cilium, situated at the tip of the retracting apical process, suggests the potential involvement of the primary cilium in RGC differentiation.

Moreover depleting critical genes like *ift88* and *elipsa*, which are responsible for primary cilia formation and maintenance, results in a noticeable reduction in the volume of zn8-positive RGCs in double morphants. This reduction leads to a smaller ganglion cell layer and a thinner optic nerve in comparison to control embryos. Therefore, these findings indicate the dual role of primary cilia in progenitor cell proliferation and maintenance as well as in the process of neurogenesis.

Other recent studies indicate that in adult mice, most RGCs possess primary cilia marked by adenylate cyclase 3 (AC3), suggesting a significant role in postnatal development and maintenance of RGC homeostasis. AC3 regulates cyclic AMP (cAMP) levels within cilia and downstream signaling pathways. Prior research has highlighted the importance of cAMP signaling in neuronal survival and axonal growth. Depletion of AC3 in olfactory neurons disrupts the formation of glomeruli and the proper projection of olfactory axons in mice.

Another commonly used marker to identify primary cilia in the brain and retina is ADP-ribosylation factor-like protein 13B (ARL13B). Mutations in the Arl13b gene have been linked to Joubert syndrome, a type of ciliopathy characterized by defects in the cerebellum, brainstem, and retinal dystrophy. In rodents, Arl13b plays a crucial role in developing the outer segment of photoreceptors and overall eye formation. The absence of Arl13b leads to premature degeneration of photoreceptors. In human embryonic stem cell-derived retinal organoids showed that during the later stages of retinal organoid development, amacrine cells consistently express ARL13B-positive primary cilia, whereas the ciliation of RGCs significantly decreases (Ning et al. 2023). Furthermore, there was a notable reduction in the number of RGCs with AC3-positive primary cilia in these organoids compared to the adult human retina. This discrepancy suggests a potential role for AC3 in the maturation and survival of RGCs. Notably, a significant number of ciliated RGCs within retinal organoids exhibit the presence of just one of the typical cilia markers, ARL13B. This carries an important implication: since RGCs in the organoids lack AC3, many of them do not possess primary cilia at all, which subsequently leads to their inability to thrive within the organoids. This outcome arises from the pivotal role of AC3 in maintaining RGC homeostasis.

Furthermore, research on the ultrastructure of primary cilia in different subtypes of RGCs showed that ARL13B and AC3 are differentially localized in the intrinsically photosensitive RGC subgroups: ON–OFF directionally selective ganglion cells have ARL13B and AC3 in their primary cilia, and alpha-RGCs primarily express AC3 in their primary cilia (Gao et al. 2022; Kowal et al. 2022).

While growth factors exhibit significant effects on developing neurons, their capacity to stimulate axon regeneration in the adult central nervous system (CNS) remains limited. A recent study employed AAV2 viruses to induce Lin28 overexpression in the retina in an effort to enhance the responsiveness of mature neurons to growth factors through dedifferentiation (Zhang et al. 2019). Interestingly, Lin28-treated retinas exhibited a regenerative response in RGC axons following injury when exposed to insulin-like growth factor-1 (IGF1). Surprisingly, this effect was not cell-intrinsic; Lin28 expression was essential solely in amacrine cells. Further investigation showed that optic nerve damage led to abnormal activity in amacrine cells, which in turn caused decreased electrical activity in RGCs and suppressed growth factor signaling. Silencing amacrine cells or using pharmacological agents to block inhibitory neurotransmission also rendered RGCs responsive to IGF1. Notably, RGCs undergoing regeneration in response to these interventions exhibited localization of IGF1 receptors on their primary cilia, which preserved their signaling competence and regenerative potential. Consequently, these findings reveal a circuit-based mechanism governing CNS axon regeneration and emphasize the significance of primary cilia as a pivotal signaling center in the regenerative process.

Moreover, the precise localization of insulin-like growth factor 1 receptor (IGF1R) within RGCs, mainly in the primary cilia, is disrupted following optic nerve injury. The manipulation of amacrine cells sustains the presence of IGF1R in the primary cilia of regenerating RGCs, and the removal of primary cilia diminishes the regenerative capacity of these treated RGCs. This suggests that signaling mediated by growth factors after cellular damage can be negatively regulated by presynaptic neurons. To assess the necessity of IGF1R concentration in the cilia, a conditional knockout of intraflagellar transport protein 88 (IFT88) was employed. IFT88 is a pivotal transport protein responsible for the formation and maintenance of primary cilia. This indicates that primary cilia in RGCs play a crucial role in facilitating regenerative responses to IGF1 (Zhang et al. 2019).

### Amacrine cells

Amacrine cells are the intrinsic interneurons of the inner retina (Fig. 1C) and are considered the most heterogeneous class of neurons within the retina. Typically, amacrine cells receive synaptic input from bipolar cells and other amacrine cells and transmit information to amacrine and ganglion cells while also providing feedback to bipolar cells (Grünert and Martin 2020). More than 30 morphologic subpopulations of amacrine cells have been characterized within the retina, and the majority function as inhibitory interneurons that release inhibitory neurotransmitters such as gamma-aminobutyric acid (GABA) or glycine (Franke and Baden 2017). The recently identified vGluT3 amacrine cell exhibits the dual release of glutamate and glycine neurotransmitters and is known to exhibit responsiveness to both object and imagined motion stimuli (Kim et al. 2015). During postnatal eye development in mice, cilia are present in Pax6-positive amacrine cells, a phenomenon also observed in primate retinas. Moreover, different subtypes of amacrine cells exhibit different cilia profiles. Unexpectedly, the elimination of primary cilia in vGluT3 amacrine cells of mice did not cause any significant visual impairments in retinal function (Ning et al. 2021). Additionally, amacrine cells have been observed to express cilia in cat and rabbit retinas through the Richardson silver stain technique (Boycott and Hopkins 1984). Further studies are necessary to clarify the specific roles of primary cilia in amacrine cells, particularly in maintaining adult retinal homeostasis and during development.

### Bipolar cells

Bipolar cells are neurons that bridge all retinal circuits (Fig. 1C) by receiving synaptic input from photoreceptors and relaying visual information to amacrine and ganglion cells (Euler et al. 2014). Bipolar cells have been found to possess primary cilia in guinea pig, mouse and human retinas when examined using electron microscopy (Ning et al. 2021).

### Horizontal cells

Horizontal cells are a distinct class of interneurons located inside the retina (Fig. 1C). Their main function involves facilitating lateral inhibition, a process that serves to enhance contrast and refine the boundaries of visual stimuli. Horizontal cells oversee the transmission of visual information between photoreceptor cells and bipolar cells in the retina, enabling effective communication (Boije et al. 2016).

Horizontal cells are generally not classified as sensory cells, and there is little evidence supporting the presence of primary cilia in horizontal cells of human retinal organoids and mice (Ning et al. 2021, 2023). Furthermore, the disruption of Hedgehog signaling, a pathway that is mostly dependent on the presence of primary cilia in mammals, in retinal progenitor cells does not appear to have any significant effects on the development of horizontal cells (Sakagami et al. 2009). However, conflicting evidence from an earlier study (Hinds and Hinds 1974) claimed that every cell of the mouse retina undergoes the creation of an axoneme at a certain stage. Given these discrepancies, further study is necessary to definitively determine whether horizontal cells in the retina have primary cilia.

### Photoreceptors

The mammalian retina harbors three types of photosensitive cells, i.e., rods, cones (containing the photosensitive opsins rhodopsin and iodopsin), and intrinsically photosensitive retinal ganglion cells (ipRGCs, containing melanopsin). While rods and cones are essential for the formation of visual images (Fig. 1B), ipRGCs receive input from rod and cone and are essential in regulating non-image-forming visual processes such as circadian rhythms and pupillary reflex (van der Merwe et al. 2018).

Photoreceptors, specifically, rods and cones, have three distinct cellular compartments: the cell body, inner segment (IS), and outer segment (OS). The inner and outer segments are connected by a nonmotile connecting cilium (CC), which is crucial for the proper function and structural integrity of these cells (Fig. 1B) (Horst et al. 1990). The outer segment and connecting cilium are often collectively referred to as specialized and modified “sensory cilia”, key components in the phototransduction pathway (Ran and Zhou 2020; Khanna 2015). The cytoskeleton of the photoreceptor sensory cilium includes the axoneme, basal body, transition zone (also called the connecting cilium), and daughter centriole. The axoneme begins at the basal body and passes through a transition zone and into the OS. The OS is densely packed with hundreds of membranous discs that are highly enriched with proteins essential to the phototransduction cascade, such as rhodopsin, arrestin, transducin, and visual pigment opsin (a G-protein coupled receptor) (Khanna 2015). These structural components highlight the functional specialization of rods and cones; rods excel in dim-light conditions while cones are responsible for color vision and function best in daylight. The IS contains cellular organelles like the endoplasmic reticulum, Golgi apparatus, alongside numerous mitochondria to meet the the high metabolic demands required for visual processing. Photoreceptor cilia are among the largest of mammalian cilia and, like other cilia lacking protein synthesis machinery, all proteins are synthesized in the IS first and then transported to the OS (Taub and Liu 2015). Approximately 10% of the OS shed daily from the distal tip and replaced by new disc formation at the base of the photoreceptor sensory cilium. This rapid turnover requires a highly efficient system for protein synthesis in the IS and effective transport of specific proteins to the OS.

All proteins that participate in sensory cilia function, as well as proteins that build the photoreceptor cilia structure, are in effect cilia proteins. Almost 25% of all retinal degeneration is caused by proteins associated with photoreceptor cilia structure and/or function. It can begin in the first or second decade of life, frequently presenting with early symptoms such as nyctalopia and loss of peripheral vision due to sensory cilia malfunction and photoreceptor cell death in the peripheral retina. The disease frequently advances to include loss of central vision as well. Functional defects, when confined primarily to photoreceptor cilia, result in nonsyndromic ciliopathies, such as Leber congenital amaurosis (LCA) and retinitis pigmentosa (RP), with occasional manifestations of ciliary dysfunction in other cell types. Pathologies connected to photoreceptor cilia also manifest as part of a broad range of syndromic ciliopathies with involvement of multiple cell types and tissues; these include Bardet–Biedl syndrome (BBS), Senior Løken syndrome, Joubert syndrome (JBTS) and Alström syndrome (AS).

### Other ciliated cell types in the retina

The inner nuclear layer (INL) of the retina comprises three distinct types of neuronal cells, including horizontal, bipolar, and amacrine cells, as well as a single class of glial cells known as Müller cells. In mice, Müller glia serve as retinal stem cells and are capable of regeneration (Das et al. 2006; Goldman 2014), proving essential for the regeneration of neuronal lineages following retinal damage. An intriguing revelation is that a subset of Müller glia possesses cilia, and the length of these cilia varies during retinal development (Ennis and Kunz 1986; Ferraro et al. 2014). This variability suggests that primary cilia may play a major role in controlling the regenerative mechanisms facilitated by Müller glia, since their role as gatekeepers of the cell cycle is well recognized (Ning et al. 2021, 2023).

The retinal pigment epithelium (RPE) is located in the outermost area of the retina and is made up of cells that contain high concentrations of pigment particles, such as melanin and lipofuscin. These pigments protect the retina from any damage caused by light exposure (Lakkaraja et al. 2020). During the development of the RPE in mice, the formation of primary cilia starts at E14.5. Subsequently, the proportion of cells possessing cilia reaches its peak (70%) 2 days later (E16.5), corresponding with the achievement of the longest ciliary length. Cilia resorb at E18.5 with a decreased length. In the RPE layer of postnatal mice, primary cilia exhibit complete maturation, characterized by the formation of a cohesive layer consisting of cubic cells arranged in an orderly manner. Furthermore, only a small percentage of cells have short primary cilia (Sun et al. 2021).

## The role of cilia in retinal neurodegenerative diseases

**Fig. 2:**
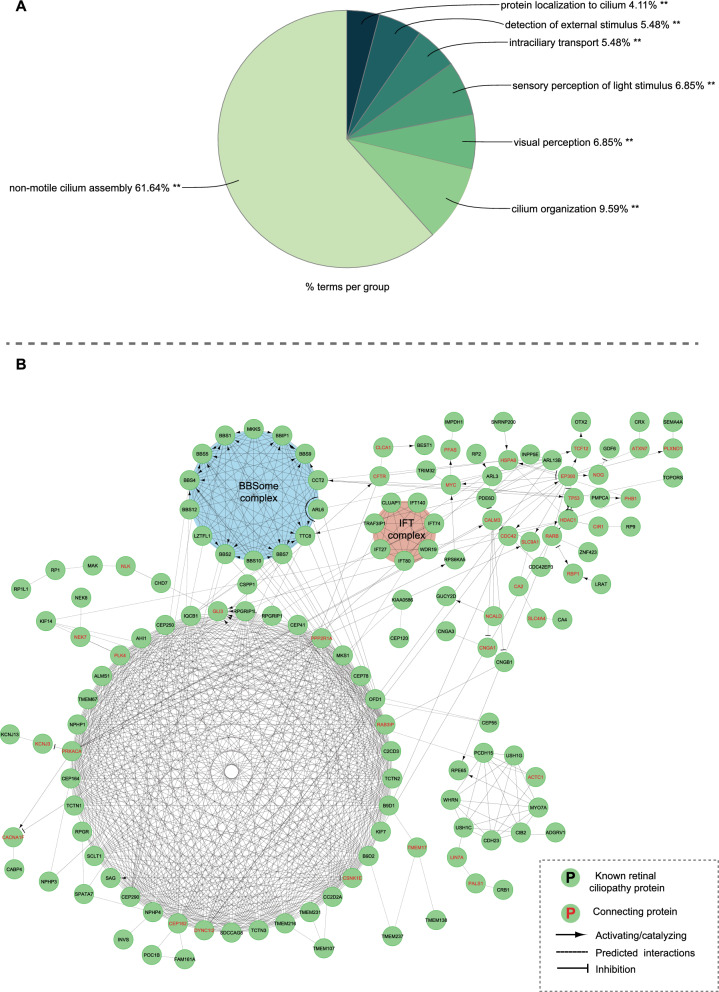
Genes associated with retinal ciliopathies. **A** Gene Ontology enrichment analysis of retinal ciliopathy genes shows that most of these genes belong to 7 functional groups. **B** A functional interaction network obtained from the reactome (https://reactome.org/) was built based on the interactions between retinal ciliopathy proteins

The contribution of primary cilia to the pathogenesis of retinal neurodegenerative diseases is an active area of research. Over 120 genes have been identified as being associated with retinal ciliopathies (Supplementary Table 1), with new genes constantly being identified. Most of these genes are related to primary cilia assembly. More specifically, transition zone/basal body genes participate in cilium organization and maintenance of transition fibers and the basal body, which act as anchors for the cilium to the cell membrane (Yuan et al. 2015; Tereshko et al. 2022). The transport of proteins and other molecules along the ciliary axoneme relies on IFT genes. Centrosome/pericentriolar material genes are involved in ciliogenesis and cilia stability. Ciliary membrane genes contribute to the structure and function of the ciliary membrane itself (Fig. 2A).

Importantly, some proteins may have multiple subcellular localizations within cilia, performing distinct functions in different parts of the organelle. Additionally, the proteins encoded by these genes are directly responsible for changes mediated by ciliopathies. All these proteins are linked to each other in a global functional interaction network (Fig. 2B); thus, the impact of a ciliopathy mutation is not restricted to a specific gene product. It also affects the interacting partners and, as a result, can impact the activity of a whole subnetwork. Instead of focusing on particular genes or loci implicated in ciliopathies, functional interaction-based analyses highlight the connections that are most changed by the disease state, helping to uncover higher-order relationships in the genetic architecture of ciliopathies.

Retinal ciliopathies can be inherited through three distinct inheritance patterns: X-linked recessive, autosomal recessive, or autosomal dominant. Since axoneme structure is shared between sperm flagella and photoreceptor cilia, individuals diagnosed with X-linked ciliopathies usually exhibit abnormalities in the morphology of sperm tails as well as impaired sperm motility. Furthermore, conditions can be categorized as nonsyndromic or syndromic. Nonsyndromic retinal ciliopathies, such as LCA and RP, are generally characterized by localized retinal defects, lacking the accompanying systemic symptoms observed in syndromic variants. Syndromic retinal ciliopathies exhibit retinal involvement as well as other systemic or organ-specific characteristics. These additional attributes have the potential to impact several physiological systems, resulting in the manifestation of a group of distinguishing characteristics (syndromic), such as BBS, JBTS and AS.

### Nonsyndromic retinal ciliopathies

#### Retinitis pigmentosa (RP, MIM 268000)

RP is an inherited retinal disorder characterized by the gradual loss of photoreceptor cells that leads to irreversible visual impairment (Verbakel et al. 2018). Individuals diagnosed with RP typically first exhibit symptoms of nyctalopia, followed by progressive loss of peripheral vision, ultimately leading to severe visual impairment or complete blindness. The clinical presentation of RP exhibits variability depending on the age at which symptoms first appear and the speed at which the degeneration of photoreceptor cells occurs. The estimated frequency of RP is approximately 1 in 3500–4000 individuals. To date, around 200 genes have been identified as being linked to RP (Shivanna et al. 2019). Among these, a minimum of 18 genes are responsible for encoding proteins that localize within the cilia of photoreceptor cells. Moreover, these genes are connected to a specific inheritance pattern: specifically, genes such as ARL6, BBS1, BBS9, C2ORF71, C8ORF37, CLRN1, FAM161A, MAK, TTC8, TULP1, USH2A, and CEP290 are linked to autosomal recessive RP; RP1, TOPORS, and RP1L1 cause autosomal dominant RP; and X-linked RP is connected to mutations in genes such as OFD1, RP2, and RPGR. The proteins encoded by these genes vary in function, and mutations at these levels alter the structure or signaling pathways of primary cilia, resulting in the demise of photoreceptor cells and vision loss.

#### Leber congenital amaurosis (LCA, MIM 204000)

Leber congenital amaurosis (LCA) is commonly characterized by an extremely early onset of visual impairment, nystagmus, and amaurotic pupils (Huang et al. 2021). LCA is a significant contributor to childhood blindness, accounting for approximately 20% of cases. LCA is considered an autosomal recessive, genetically heterogeneous condition. The discovery of de novo mutations in CRX, OTX2, and IMPDH1, which are associated with LCA, has sparked a discussion on the potential presence of dominant inheritance patterns in certain cases of LCA (Rivolta et al. 2001; Bowne et al. 2006; Zou et al. 2013; Roger et al. 2014). Approximately 75% of LCA cases can be attributed to particular mutations (Supplementary Table 1). The disease-causing mechanisms that are commonly observed include phototransduction (GUCY2D, AIPL1, RD3, and KCNJ13), retinoids (RPE65, LRAT, and RDH12), ciliary transportation (LCA5, CEP290, RPGRIP1, SPATA7, TULP1, and IQCB1/NPHP5), photoreceptor morphogenesis (CRX, CRB1, GDF6, and PRPH2), guanine synthesis (IMPDH1), and photoreceptor differentiation (OTX2). The underlying mechanism responsible for the LCA phenotype associated with recently discovered mutations in USP45 (LCA type 19) and other genes remains a subject of debate, requiring further investigation.

#### Cone-rod dystrophy (CORD, MIM 120970)

There are more than 30 distinct types of cone-rod dystrophies that can be classified as recessive, dominant, or X-linked (Tsang and Sharma 2018). One of the distinguishing features of these pathologies is the presence of retinal pigment deposits that are observable during fundus examination. These deposits are primarily concentrated in the macular region. In contrast to retinitis pigmentosa (RP), which is characterized by the initial degeneration of rod photoreceptors followed by the subsequent loss of cone photoreceptors, CORD exhibits a reverse pattern of progression.

The pathogenesis of CORD involves four primary causative genes: ABCA4, CRX, GUCY2D, and RPGR (Birtel et al. 2018). ABCA4, a protein involved in the transport of retinaldehyde, accounts for approximately 30 to 60% of autosomal recessive CORDs, with mutations at this level leading to impaired cone and rod photoreceptor function. CRX and GUCY2D are factors that play crucial roles in the development of photoreceptor cells and the phototransduction pathway, respectively; mutations in these proteins lead to impaired photoreceptor function and degeneration and are responsible for a significant number of reported cases of autosomal-dominant CORDs. Mutations in RPGR, on the other hand, lead to disruptions in ciliary function in photoreceptor cells and cause degeneration of cones and rods. These mutations are involved in approximately two-thirds of cases of X-linked retinitis pigmentosa (RP) and an undetermined fraction of X-linked CORDs. In addition, mutations in genes such as RP1 and CEP290 have been linked to certain occurrences of CORD (Supplementary Table 1).

### Syndromic retinal ciliopathies

#### Senior–Løken Syndrome (SLS, MIM 266900)

Senior–Løken Syndrome (SLS) is an autosomal-recessive disorder characterized by the presence of juvenile nephronophthisis, retinal degeneration that results in blindness, and the eventual development of end-stage renal disease (Tsang et al. 2018). Mutations in at least 13 genes have been reported to lead to SLS (Salomon et al. 2008). Most NPHP proteins have been reported to localize to the base or axoneme of primary cilia as well as to the connecting cilium of the photoreceptor (Supplementary Table 1). The presence of germline deletions in Nphp8 and Nphp12 has been found to result in embryonic lethality. Several animal germline knockouts have been produced, specifically targeting the NPHP1, -3, -4, -5, -6, -7, and -14 genes.

However, none of these loss-of-function rodent models have successfully recapitulated the entire phenotype of SLS observed in humans. Since various animals exhibit distinct processes of disease onset and the properties of organs vary according to their evolutionary history, animal disease models can have restricted or limited use. In vitro disease models can be used as another alternative for ciliopathies. A recent study employed dermal fibroblasts from patients with NPHP5-LCA, retinal pigment epithelium (RPE) cells and retinal organoids from patient-derived induced pluripotent stem cells (iPSCs) to develop a 3D culture system mimicking the in vivo model (Kruczek et al. 2022). The investigation revealed that retinal organoids displayed aberrant elongated cilia and decreased quantities of CEP290. Additionally, the photoreceptors within these patient organoids exhibited compromised protein localization and abnormal extension of outer segments.

#### Bardet–Biedl Syndrome (BBS, OMIM 209900)

Bardet–Biedl Syndrome (BBS) is a rare autosomal-recessive disorder characterized by a combination of clinical features, including retinal degeneration, obesity, postaxial polydactyly, learning impairments, renal involvement, and male hypogenitalism. BBS is genetically diverse, and 18 genes (BBS1-18) have been characterized to date. Among these genes, BBS1, BBS2, BBS4, BBS5, BBS7, BBS8, BBS9, and BBS18 have been shown to form a stable complex known as the “BBSome.” The BBSome complex functions as an adaptor between cargo and the IFT complex and directs the movement of over one hundred proteins from the OS to the IS of photoreceptors. This mechanism effectively prevents the excessive accumulation of these proteins in the OS and maintains its structural integrity. Furthermore, the absence of specific functional subunits of the BBSome leads to the malformation of the OS.

Approximately 70–80% of cases can be attributed to mutations in established BBS genes. Due to limited knowledge regarding the necessity of the BBSome during eye development and maturation, no definitive association has been established between the genotype and clinical manifestations of BBS for the majority of reported variants. Multiple studies have indicated the presence of a less severe phenotype when certain mutations in BBS genes are observed (M’hamdi et al. 2014; Berezovsky et al. 2012).

Through the examination of BBS12 mouse models, researchers discovered that the initiation of an unfolded protein response, induced by protein accumulation in the endoplasmic reticulum of photoreceptors, can stimulate apoptosis and lead to degeneration of the retina (Mockel et al. 2012).

#### Joubert Syndrome (JBTS, MIM 213300)

Joubert Syndrome (JBTS) is an autosomal-recessive disorder characterized by symptoms such as episodic hyperpnea, global developmental delays, ataxia, and aberrant eye movements. Currently, pathogenic variations in more than 40 genes have been associated with JBTS. Among these genes, most exhibit autosomal recessive inheritance, while one gene is X-linked (Parisi and Glass 1993; Dong et al. 2023). The most common genes were AHI1, CC2D2A, CEP290, CPLANE1, CSPP1, INPP5E, KIAA0586, MKS1, NPHP1, RPGRIP1L, TMEM67, and TMEM216 (Supplementary Table 1). Less common genes included ARL13B, B9D1, C2CD3, CEP104, CEP120, KIAA0556, PDE6D, POC1B, TCTN1, TCTN3, TMEM138, TMEM231, TMEM237, CHD7 and OFD1 (X-linked) (Wang et al. 2018).

#### *Alström Syndrome* (ALMS, MIM 203800)

Alström Syndrome is an autosomal-recessive monogenic disease caused by homozygous or compound heterozygous variants in the ALMS1 gene. The clinical manifestation of ALMS is characterized by a variety of clinical features, including retinal degeneration, obesity, sensorineural hearing loss, insulin resistance, and progressive hepatic and renal dysfunction. The ALMS1 gene consists of 23 exons and encodes a protein of 4169 amino acids. The expression of ALMS1 is observed in a broad range of tissues, and it is localized in the centrosomes as well as at the base of cilia. To date, more than 230 mutations in the ALMS1 gene have been identified as causative factors for various diseases (Marshall et al. 2015). A significant proportion (96%) of the observed mutations are classified as nonsense and frameshift variants, which are anticipated to result in the premature truncation of the protein. The precise function of the protein derived from the ALMS1 gene remains incompletely elucidated; however, it has been implicated in various biological processes, including ciliary function, regulation of the cell cycle, organization of microtubules, and intracellular transport.

ALMS1 mutations account for the preponderance of Alström syndrome cases. However, there are certain individuals who exhibit clinical symptoms of Alström syndrome, but the genotype–phenotype correlation in these cases remains unidentified. Researchers continue to investigate the condition in an effort to identify additional genes or genetic factors that may play a role in its development.

#### Usher Syndrome (USH, OMIM 276901)

Usher Syndrome is an autosomal-recessive syndromic ciliopathy characterized by congenital hearing impairment and the gradual development of retinitis pigmentosa in adulthood. Three distinct clinical types of Usher syndrome (USH1, USH2 and USH3) have been classified according to the severity of hearing loss, the age at which retinitis pigmentosa develops, and the presence or absence of vestibular response (Delmaghani and El-Amraoui 2022).

USH1 is the most severe subtype and is characterized by severe to profound congenital hearing loss, balance problems, and early-onset retinitis pigmentosa (RP). Genes associated with USH1 include MYO7A, CDH23, PCDH15, USH1C, USH1G, CIB2 (USH1J), and several others (Supplementary Table 1). USH2 is the most frequent subtype and is characterized by moderate to severe congenital hearing loss and retinitis pigmentosa (RP) but not vestibular dysfunction. The most common gene associated with USH2 is USH2A, accounting for a majority of cases. Other genes include GPR98 (PCDH15), DFNB31, ADGRV1 (USH2C), and WHRN. USH3 is the rarest form and is characterized by progressive hearing loss and retinitis pigmentosa (RP) but not vestibular dysfunction. Usher syndrome is caused by mutations in the CLRN1 gene, which is thought to play a role in the development and maintenance of photoreceptor cells and inner ear hair cells. Cases that do not fit into the established criteria for USH1, USH2, or USH3 are defined as atypical Usher syndrome (atypical USH). Pathogenic variants in many USH genes associated with atypical USH have been reported, including MYO7A, USH2A, CDH23, ADGRV1, CEP250, CEP78, and ABHD12.

Mutations in ADGRV1 and CIB2 have been linked to three separate subtypes of Usher syndrome. The two genes encode two proteins from independent protein families, adhesion G protein-coupled receptor (ADGRV1) and Ca^2+^- and integrin-binding protein 2 (CIB2). Interestingly, the comparison of the protein interactomes of CIB2 and ADGRV1 demonstrated a significant overlap in their interaction partners. Out of the 386 proteins identified as interaction partners of CIB2, 270 proteins have already been recognized as binding partners of ADGRV1 (Linnert et al. 2023; Knapp et al. 2022).

Furthermore, the interaction between CIB2 and ADGRV1 within a broader ciliary network that is also associated with USH, BBS, and specific forms of LCA (Fig. 2B) provides compelling evidence for shared molecular pathomechanisms underlying different syndromes (Linnert et al. 2023). This finding presents an opportunity to identify common therapeutic targets that can address the underlying defects in patients affected by these ciliopathies, regardless of the specific mutations involved.

#### Meckel–Gruber Syndrome (MKS, MIM 249000)

Meckel–Gruber Syndrome is an autosomal-recessive condition typically characterized by three symptoms: cerebral protrusion, cystic renal anomalies and polydactyly. The syndrome can also cause problems with the development of the eyes and other facial features.

To date, 17 genes have been identified as causative factors for MKS (Supplementary Table 1) (Hartill et al. 2017). Mutations in the MKS1 gene have been identified as the causative factor in approximately 7% of all MKS cases. Notably, these mutations account for approximately 70% of MKS cases specifically within the population of Finland (Hartill et al. 2017).

MKS cases display a notable degree of clinical overlap with JBTS, and at least 10 mutations (MKS1, TMEM216, TMEM67, CEP290, CC2D2A, NPHP3, TCTN2, B9D1, B9D2, and TMEM231) have been identified in both diseases.

## Current therapeutic approaches to treat retinal ciliopathies

Retinal neurodegenerative diseases caused by dysfunction or mutations in genes associated with primary cilia or photoreceptor cilia are currently incurable. Nevertheless, there are several treatments and interventions that are capable of alleviating the symptoms and delaying the advancement of the disease. While symptomatic treatment has shown efficacy in enhancing the quality of life for those with retinal ciliopathies, it does not directly address the underlying disease mechanisms, nor does it possess preventive properties.

Ciliopathy phenomena encompass a broad spectrum, ranging from specialized phenotypes that are extremely specific to certain cell types or the eyes to diverse syndromic ciliopathies that can impact the entire body. We have a limited comprehension of how a genetic abnormality affects several levels of biological organization, ranging from genotype to clinical phenotype. Given the complexity of ciliopathies and the diverse genetic and molecular causes, the therapeutic strategy for retinal ciliopathies may vary. Gene therapy has the potential to treat specific ciliopathies, and other therapeutic approaches (e.g., small molecules) may focus on targeting specific cellular processes related to cilia function.

### Gene therapy for ciliopathy

The identification of disease-associated genes and the characterization of their activities has resulted in a significant rise in the development of molecular therapies. In the case of autosomal-recessive diseases, therapies include augmentation of the right (nonmutated) copy of the affected gene, while knocking down the expression of the mutant allele is the employed strategy in the case of autosomal-dominant diseases. Current advancements in genome editing technology have also enabled precise modification of gene sequences inside the genome. The most widely employed technique for gene delivery is the subretinal injection of viral vectors, particularly adeno-associated viruses (AAVs).

Clinical investigations of RPE65 gene augmentation therapy in LCA patients (Luxturna®) showed the safety of AAV-mediated gene delivery and improved functional vision in certain individuals (Bennett et al. 2016; Russell et al. 2017). In 2017, Luxturna has become the first FDA-approved gene therapy for an inherited retinal disease, offering a viable treatment option for RPE65-related LCA. Another promising gene therapy is ProQR’s Sepofarsen (QR-110), an RNA-based therapy aimed at correcting the p.Cys998X mutation in the CEP290 gene, which is a common cause of LCA10 (Russell et al. 2022). Early clinical trials have shown promise in improving visual function in affected individuals.

In addition to these, emerging therapies such as CRISPR/Cas9 gene editing (Ran et al. 2013) and antisense oligonucleotides (ASOs) (Dhuri et al. 2020) are also being explored. CRISPR/Cas9 technology is being investigated for its potential to directly correct genetic mutations responsible for retinal ciliopathies, with several preclinical studies underway and clinical trials anticipated in the near future. This precise gene-editing method offers the possibility of a permanent cure for genetic disorders. Antisense oligonucleotides (ASOs), designed to target and modify RNA transcripts of mutated genes, are another promising approach. ASOs for various retinal diseases, including those caused by ciliopathies, are in the pipeline, with some already in early-phase clinical trials. These ASOs work by binding to specific mRNA sequences, leading to the degradation of the mutant mRNA or the correction of splicing defects (Dhuri et al. 2020).

Although current gene therapy techniques show promise in the early phases of treatment, their effectiveness decreases in the long term (Ziccardi et al. 2019). This is mainly due to incomplete knowledge of the disease process and complex protein interactome that manifest the disease.

### Small-molecule approach

Pharmacological intervention can potentially be employed as a strategy for decreasing cellular loss and tissue degeneration, serving as an interim solution until individualized remedial gene therapy is developed. While there is a clear link between genotype and phenotype, it remains difficult to find the appropriate drug target and develop small-molecule drugs for ciliopathies.

Several studies have attempted to identify potential compounds for the treatment of retinal ciliopathies by cell-based screening of chemical libraries. For example, one study used a human CEP290null RPE1 cell line generated through CRISPR/Cas9 technology mimicking mutations linked with LCA to screen 2789 synthetic and natural compounds for potential therapeutic efficacy in repairing the ciliogenesis deficiency induced by CEP290 loss. The strongest rescue effect was demonstrated by the flavonoid eupatilin and its analogs. Eupatilin, in particular, can alleviate ciliogenesis abnormalities and improve the function of the ciliary transition zone damaged by CEP290 deletion. Moreover, in rd16 mice with a Cep290 in-frame deletion, eupatilin enhanced opsin transport to the photoreceptor outer segment and retinal electrical responses to light stimulation (Kim et al. 2018). Another study used retinal organoids derived from induced pluripotent stem cells (iPSCs) of both mouse model and LCA patients to test over 6000 compounds. Reserpine was identified as a lead compound that maintained photoreceptor development and survival (Chen et al. 2023). Reserpine works by restoring the balance between autophagy and the ubiquitin–proteasome system and improving primary cilium assembly, showing promise as a potential therapeutic for LCA10. Both studies underscore the utility of drug repurposing to expedite the development of treatments for retinal ciliopathies like LCA. Proteins that interact physically with one another generally participate in similar biological processes and contribute to similar organismal properties, in both a healthy state and the presence of ciliopathies (Fig. 2B). A protein interactome, which includes the entire network of protein interactions, is critical in bridging the gap between genotype and phenotype. Existing systematic protein network techniques imply that small molecules treat diseases by targeting proteins in a network of physical interactions that are close to disease proteins. However, small molecules can also treat diseases by targeting distant proteins that impact the same biological functions.

## Conclusion

This review aims to elucidate the role of cilia in the different retinal cell subtypes, drawing from recent evidence linking retinal neurodegenerative diseases to defects in retinal primary cilia. Besides these diseases, the broader impact of neurodegenerative conditions such as Parkinson’s disease (PD), Alzheimer’s disease (AD), and Amyotrophic Lateral Sclerosis (ALS) on retinal primary cilia has also emerged as a significant area of study (Ma et al. 2022). These disorders show how primary cilia in the retina might be impaired in their role of mediating critical signaling pathways, primarily due to the neurodegenerative processes inherent in each disease. Specifically, in the context of AD, retinal primary cilia could exhibit abnormalities in both structure and function, likely resulting from the neurotoxic environment created by amyloid deposits and neurofibrillary tangles (Vorobyeva and Saunders 2018).

The investigation of these diseases underscores the necessity for advanced research to fully understand the specific changes occurring in retinal primary cilia. Developing more sophisticated imaging techniques to observe these tiny structures in vivo, along with experimental models that accurately mimic human disease processes, are critical steps towards this goal. As we dive deeper into the genetics underlying these conditions, it is important to recognize the heterogeneity within them. Multiple genes can be associated with a single disorder, and while the genetic mutations discussed here represent some of the most well-known associations, other genes linked to different ciliopathies or syndromes may also play significant roles in retinal neurodegenerative disorders.

Given this complexity, individuals diagnosed with retinal neurodegenerative diseases stemming from primary cilia or photoreceptor cilia dysfunction must adopt a personalized treatment plan. This plan should be developed in close collaboration with a multidisciplinary team of healthcare professionals, including ophthalmologists, geneticists, and other specialists. As research continues to advance, especially in the fields of genetic therapies and ciliopathy-related remedies, the prospect of more targeted and effective therapeutic options becomes increasingly feasible, promising new hope for affected individuals. This continuous evolution in our understanding and treatment capabilities reflects the dynamic nature of the field and the critical role of ongoing research.

### Supplementary Information

Supplementary Material 1. Table S1. The list of retinal ciliopathies genes reported in literature.

## Data Availability

All data generated or analyzed during this study are included in this published article and its supplementary information files.
